# Interprofessional pain education—with, from, and about competent, collaborative practice teams to transform pain care

**DOI:** 10.1097/PR9.0000000000000663

**Published:** 2018-05-30

**Authors:** Debra B. Gordon, Judy Watt-Watson, Beth B. Hogans

**Affiliations:** a Division of Anesthesiology and Pain Medicine, University of Washington School of Medicine, Seattle, WA, USA; b Lawrence S. Bloomberg Faculty of Nursing, Massey College, University of Toronto, Toronto, Ontario, Canada; c Department of Neurology, Johns Hopkins School of Medicine, Baltimore, MD

**Keywords:** Interprofessional education, Pain competency, Collaborative practice

**Key Points**

1. A competent, collaborative, interprofessional team centered on the patient is necessary for quality pain care; however, interprofessional collaborative practice is not yet an integral part of all health professions education programs.
2. Interprofessional education involves 2 or more professions learning “with, from, and about” to enable effective collaborative practice and improve health outcomes.
3. Core competencies and curricular resources are available for interprofessional education and pain and can be adapted for use at all levels of health professions education.

## 1. Introduction

Pain is a complex experience that impacts health, productivity, and well-being. It requires a collaborative team approach with a common language and clear understanding of roles and responsibilities. With few exceptions, a minimum amount of pain content has been documented in health sciences curricula, and much of that has been fragmented by profession and delivered within a crowded agenda of conventional course topics such as anatomy and physiology.^21^ Most health professionals learn pain management on the job and are often ill-prepared to function as a team member in the real world. Despite documentation of the need for improved education on pain of all types, consistent professional training in pain is not widespread and innovation is warranted. The 2018 IASP Global Year for Excellence in Pain Education is a call to action on multiple levels. The purpose of this report is to describe opportunities for mutual learning through interprofessional (IP) pain education. Interprofessional education (IPE) is a growing trend across health professions and has been defined (Table 1) as when 2 or more professions learn with, from, and about each other to improve collaboration and the quality of care.^16^ For IP learning to occur, all 3 “with, from, and about” must be present.^11^

**Table 1:**
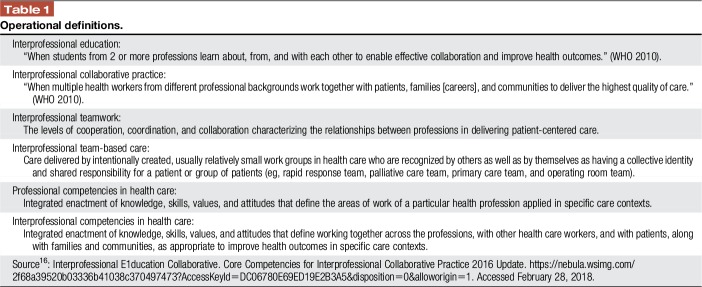
Operational definitions.

## 2. With others–learning together to facilitate interprofessional collaborative practice

The complexity of health care across the globe, technological advances, and modern models of care delivery has created demand for a practice-ready workforce and effective teamwork.^31^ Medical errors often result from poor communication within and across teams; high functioning teams improve outcomes of care.^18^ Professional education has not kept pace with increasing demands for collaboration-ready health workers in part because of disjointed, outdated, and static curricula; furthermore, a glaring mismatch of competencies to patient and population needs persists.^12^ We educate students most often in uniprofessional settings (silos) with little opportunity to learn and practice together. Although learning experiences in the clinic or on a ward offer more opportunities to learn with, from, and about other health care professionals, there may be few role model IP teams in the real environment. Uniprofessional education is inadequate to prepare health care trainees to work in teams and can spur competition rather than cooperation between the professions.

Recognizing this struggle, the World Health Organization (WHO) issued a *Framework for Action on Interprofessional Education and Collaborative Practice* in 2010.^34^ The report contextualizes existing health systems, commits to implementing principles of IPE and collaborative practice, and champions the benefits of IP collaborations with regional partners, educators, and health workers. Contemporaneously, the Lancet Commissions issued a foundational report^12^ developed by 20 health profession leaders from diverse countries advancing a common strategy for educational reform in medicine, nursing, and public health. The Lancet Commission calls for education reform that is guided by the desired outcomes of transformative learning and interdependence in education. Transformative learning involves fundamental shifts from fact memorization to synthesis of information for decision making; from seeking professional credentials to achieving core competencies for effective teamwork in health systems; and adaptation of global resources to address local priorities.^12^ Interdependence stresses the system approach that offers insights into the dynamic and nonlinear nature of a complex system that cannot be gained by studying components of the environment in isolation. This report also underscores the pace, scale, and intensity of globalization impacting interactions of health systems and education.

In several countries, collaboration of national associations of health profession regulatory bodies has given rise to recommendations for core competencies for IP collaborative practice designed to guide curriculum development in interactive learning. For example, competencies^16^ developed by the Interprofessional Education Collaborative (IPEC) in the United States have become part of the global conversation (Table 2). The Global Forum on Innovation in Health Professional Education has hosted a series of meetings engaging stakeholders and policymakers through linked projects and networks in Uganda, South Africa, India, and Europe.^8^ Updated in 2016, the IPEC competencies integrate explicit population health outcomes with individual care competencies to form an expanded model that targets desirable health system goals. Interprofessional collaboration, in this framework, is the central domain under which the original competencies are arranged. Similarly, the Canadian IP Health Collaborative developed the *National Interprofessional Competency Framework* in 2010, which has been used in various countries and academic settings.^5^

**Table 2:**
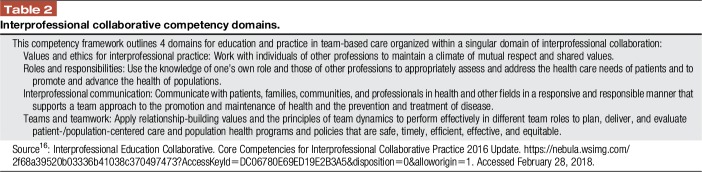
Interprofessional collaborative competency domains.

Interprofessional collaboration occurs when learners/practitioners, patients/clients/families, and communities develop and maintain working relationships that enable optimal health outcomes.^5^ Interprofessional collaborative practice occurs when multiple health workers from different professional backgrounds work together with patients, families, and communities to deliver the highest quality of care. Interprofessional core competencies build on modern educational theory and practice to bring together all health professions with shared language, vision, and goals (Table 3). These competencies are important for positive outcomes, including those we aim for in quality pain care.

**Table 3:**
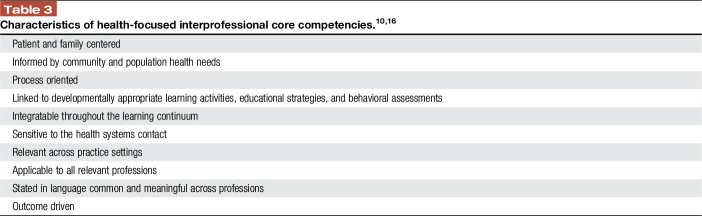
Characteristics of health-focused interprofessional core competencies.^10,16^

## 3. From others—learning from different professions to facilitate collaboration and communication

Collaborative approaches are invaluable when pain management is complex, requiring the knowledge and skills of more than one profession. It is logical then, that to work together, future health care workers would benefit from learning together to understand each other's roles and responsibilities and how to communicate using common language. The provision of opportunities for student interaction is fundamental to the learning experience to develop an understanding of the perspective of various professions and to foster a climate of mutual respect and relationship-building values. Interprofessional education requires active learner participation and case-based content that is authentic and foundational to many health professionals.^6,26^ Although most IPE is focused on prelicensure students, literature is emerging in post graduate clinical education. Themes in the context of back pain in a primary care setting included the context, value of involving the patient, listening, time and learning together.^7^ The intent is to impact practice and improve quality of health care.

## 4. About others–attaining competence to use knowledge of one's own role and those of other professions to address pain care needs

Pain experience is multidimensional; therefore, pain education draws on not only mechanisms but also a variety of theories such as relational, professionalism, and social constructivism and is grounded in adult learning theory.^31,33^ The concept of communities of practice and situated learning is also important as students move from learning about their own profession to other professions and members of a team.^31^ These concepts reinforce a model of multiprofessional team management of pain championed long ago by John Bonica. Learners need to become self-directed, critical thinkers and reflective practitioners, able to function as members of teams, and be good communicators, adaptable to change and continuing to learn through professional experiences.^3,22^ Interprofessional education is not a replacement for education specific to each profession, a reason to lose individual professional identity, the only innovation needed in the health system, and an end in itself. We do not do IPE for its own sake; we do it to help understand each other's roles and contributions to work together in a real-world practice setting.

## 5. Barriers to overcome

A number of significant barriers must be overcome to successfully implement and sustain a culture of IPE.^22,32^ Leadership at the highest level is needed for a culture change to be successful. For example, licensure and accreditation requirements do not currently reinforce preparation for collaborative practice in most countries. A survey of 41 countries from WHO's 6 regions representing various income economies reported IPE was often voluntary.^27^ Moreover, the lack of compulsory IPE and pain competencies for entry-to-practice graduates has implications for advancing skillful and ethical practice; it can limit the capacity of health care professionals to alleviate suffering, foster autonomy, and use resources justly.^32^ As well, many faculty are trained and familiar with the didactic teacher role rather than how to be an effective IPE facilitator and are not comfortable teaching pain content^4^; faculty education and development are needed. Faculty and clinician composition in the development and implementation of IPE activities may influence the outcomes of the learning activities.^25,33^ Evidence is scarce in developing countries, but challenges may be similar including curriculum structure and complexity. It has been suggested that barriers be taken as opportunities to transform approaches to core health problems in developing countries.^30^

Modifications in physical classroom space, competition for curriculum hours, and coordination of schedules can be challenging. Ideally, students should be introduced to IPE early with learning activities that build on competencies. Curriculum design is an iterative process necessitating modifications of complexity in patient cases and also the challenges of integrating clinical content to meet the needs of all levels of learners.^33^ Perceived differences in hierarchy, power status, and unequal participation rates among certain health professions have also been described as challenges.^24^ However, IPE can provide an opportunity to transform the way we socialize students by improving the understanding and respect of each other's unique roles and responsibilities within the team. Of course, the ultimate challenge is to harmonize learning experiences with well-functioning IP teams in clinical practice. Involvement of clinicians in curriculum development and implementation can help to insure real-world pain care and patient-centered modeling.^33^

## 6. Defining components of interprofessional education–competencies (learning outcomes), curriculum (learning plans), and content (learning objectives)

**Figure 1.**
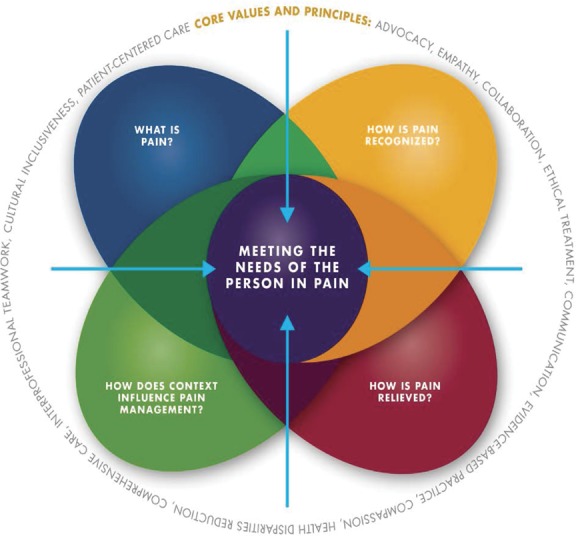
Core competencies for pain management. These core competencies pain assessment and management were developed through an interprofessional consensus process^10^ to address prelicensure pain management education in all major health care professions that are consistent with the IASP pain curricula outlines. Graphic created by Ian Koebner, PhD. Used with permission University of California Regents or Graphic courtesy of University of California Regents.

Competency is the desired outcome of education. Distinct from learning objectives that emphasize gains in factual knowledge, attitudes, and skills, competency places emphasis on students' capacity to act effectively in relevant clinical situations.^10^ Competency generally includes observable phenomena such as being able to demonstrate the ability to explain a treatment or educate a patient about relevant treatment adverse effects. It also includes appropriate attitudinal and affective qualities to the extent that such are observable, eg, being able to maintain perceptibly compassionate communication while examining a painful part, potentially gauged through the use of interpersonal skills checklists. Core competencies in pain management for health professional education have been established.^10^ These pain competencies address the fundamental concepts and complexity of pain; how pain is observed and assessed; collaborative approaches to treatment options; and application of competencies across the life span in the context of various settings, populations, and care team models (Fig. 1). A set of values and guiding principles is embedded within each domain. These competencies can serve as a foundation for developing, defining, and revising curricula and as a resource for the creation of IP learning activities across health professions designed to advance care that effectively responds to pain.

Pain curricula outlines provide the template that helps to structure learning. Curricula include considerations of sequencing material, developmental appropriateness, and coordination of different health professions' learning activities, so that students from different health profession programs will learn about for IPE at the same time. The *IASP Pain Curriculum Outlines*^15^ provide recommended curricula for pharmacy, psychology, physical therapy, occupational therapy, nursing, medicine, dentistry, social work, and IPE. Each is arranged to address 4 main domains and related core competencies including (1) the multidimensional nature of pain, (2) pain assessment and measurement, (3) the management of pain, and (4) pain in specific clinical conditions. The outlines are helpful for establishing courses that provide an integrated foundation in pain at both the undergraduate and graduate levels. With this foundation, students are prepared to understand and approach patients with many forms of pain, as well as provide support to families and caregivers. All IASP curricula outlines including IPE were updated in 2017 for the Global Year for Excellence in Pain Education.

Content is the description of what is being taught at the most granular level, eg, what are the learning objectives. Content serves as an important common language necessary to effectively communicate with each other about the specific elements of our uniprofessional and IP learning plans.

Three teaching modules that address a number of IASP topics and are adaptable for IPE are available on the Portal of Geriatric Online Education (POGOe.org).^20^ In the United States, the National Institutes of Health has created a freely accessible portal of pain education online learning modules. Based on a variety of local models of IP collaboration, these modules demonstrate that IPE can take various forms depending on the specific professions engaged and the goals for learning.^23^ A unique and perhaps most comprehensive program is the 20-hour University of Toronto's Pain IP Curriculum involving students from 7 professional programs. The program's design and implementation components are described in the Pain IP Curriculum Model as (1) dynamic, (2) competency-based, (3) interrelated, and (4) collaborative with the patient focus at the center.^33^ Experience with the program has informed the creation of an eLearning Pain Education Interprofessional Resource that is internet accessible and available on request.^19^ As a blended eLearning program, Pain Education Interprofessional Resource has been designed as a self-learning resource to be coupled with facilitated small group, IP, collaborative discussion.

## 7. Outcomes of interprofessional education

Measuring outcomes of IPE can be quite challenging. Large gaps regarding methods, theory, and context remain, and most studies focus on short-term results. The heterogeneity of contexts, variety of interventions, and methodological limitations makes it difficult to draw generalizable inferences about key elements and effectiveness of IPE.^17,26^ Evaluation should ideally link to clinical practice, but there is a paucity of contextually and synthesized literature regarding outcomes, particularly for pain management.^24^ University-based IPE using patient scenarios and group work in small teams, as contrasted to didactic lectures, has been shown to be feasible and has led to improved attitudes toward IP interaction and teamwork and improved understanding of health professional roles.^24^ Studies of IPE have found differences between professions, with students in professions deemed psychosocial were more positive about IPE than students in biomedical career tracks.^13^ Similarly, Erickson et al.^9^ found that IP mentorship and group participation improved first year medical students' pain management skills but did not have the same effect on fourth year nursing student performance. Differences were attributed in part to experience in clinical settings but also suggested that combining different levels of students is acceptable if they are of similar age and life experience. A significant positive shift in the pain knowledge and attitudes toward collaboration has been demonstrated through IPE.^14,28^ Simko et al.^29^ reported an increased knowledge and understanding of the importance of other profession's role in pain management in an IPE course for nursing and pharmacy students. Other studies have reported high student satisfaction and significant improvement in self-efficacy^1^ as well as respect for each other's roles and responsibilities.^2^ Positive changes have also been reported in pain assessment and documentation behaviors from IPE.^17^

## 8. Summary

The delivery of effective pain management can be complex, requiring collaborative, team approaches that exceed the expertise of any one profession. Interprofessional collaboration is increasingly recognized as a core skill for all clinicians and is beginning to be required by some accrediting bodies for medical, nursing, pharmacy, physician assistant, and social work programs. However, IP collaborative practice is not yet an integral part of all health professions education programs. Recommendations of the WHO^34^ and other leading organizations recognize IP collaborative practice and education as a central component of transformative improvements in health care. Based on work in a number of global settings, recommendations for educational change to incorporate IP collaboration into practice are available and undergoing further development.

Creating IPE learning opportunities is important. The intent of IPE is to produce a collaborative practice-ready workforce to improve the quality of health care. Students should be introduced to IPE early and have developmentally appropriate opportunities throughout a curriculum program. Students can change agents in the real world to continuously improve the way health professionals work together, mentor students and improve the quality of pain care. The quality and rigor of IPE research is inadequate, and research needs to move beyond feasibility and attitudes toward long-term improvements in clinical care.

When focused on pain, IPE is likely to provide substantive benefits in the real-world practice setting, but barriers to IPE adoption, including slow adoption of pain-focused competencies and cultural habits, limit uptake. When able to overcome these obstacles, IPE has the capacity to harmonize learning experiences and promote patient-centered socialization of health profession trainees at all levels. Importantly, communicating and assessing innovation in IPE relies on understanding the conceptual education framework built on key elements of competencies (learning outcomes), curriculum (learning plans), and content (learning objectives). Although more work is needed to identify the most effective approaches, and even fundamentally to define meaningful approaches to outcomes assessment, models of education such as IP workshop training and online education exist with positive impact. We leave readers with a brief table of actions they can take to advance and transform health professions' education (Table 4).

**Table 4:**
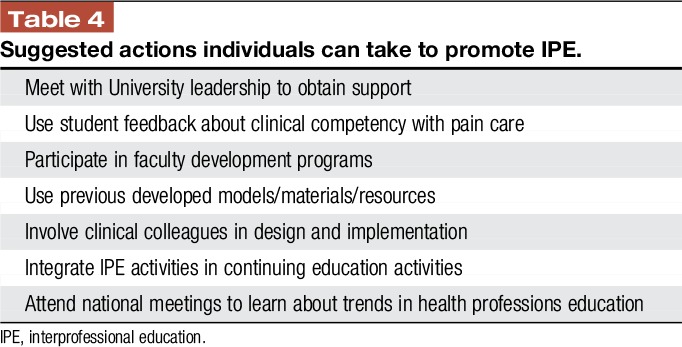
Suggested actions individuals can take to promote IPE.

## Disclosures

The authors have no conflict of interest to declare.

D.B. Gordon and B. Hogans hold positions of leadership in their University's NIH Pain Consortium designated Centers of Excellence in Pain Education (CoEPEs). J.Watt-Watson is a principal leader in the University of Toronto's IP curriculum program.
